# Tuberculous disseminated lymphadenopathy in an immunocompetent non-HIV patient: a case report

**DOI:** 10.1186/1752-1947-3-9316

**Published:** 2009-12-03

**Authors:** Irini Gerogianni, Maria Papala, Konstantinos Kostikas, Maria Ioannou, Argiroula-Vasiliki Karadonta, Konstantinos Gourgoulianis

**Affiliations:** 1 Department of Respiratory Medicine, Medical School, University of Thessaly, Larissa 41110, Greece; 2 Department of Pathology, Medical School, University of Thessaly, 41222 Larissa, Greece

## Abstract

**Introduction:**

In cases of patients with disseminated lymphadenopathy, the differential diagnosis has to include both benign and malignant causes, including sarcoidosis, metastatic disease, lymphoma and, although rarely present, tuberculosis. Tuberculosis is still one of the most frequently occurring infectious diseases worldwide. However, disseminated mycobacterial lymphadenitis is rare in immunocompetent patients.

**Case presentation:**

We present the case of a 56-year-old Caucasian Greek male, who was immunocompetent and HIV negative, with a two-month history of recurring fever, loss of appetite and disseminated lymphadenopathy. The patient was diagnosed with mycobacterial lymphadenopathy.

**Conclusion:**

This case highlights the need for suspicion in order to identify mycobacterial infection in patients with generalized lymphadenopathy, since misdiagnosis is possible and may lead to fatal complications for the patient.

## Introduction

Disseminated lymphadenopathy presents a diagnostic dilemma and the differential diagnosis has to include tuberculosis (TB), although rarely present. TB is still one of the most frequently occurring infectious diseases worldwide. According to the World Health Organization, approximately one third of the world's population is infected with tubercle bacilli. Eight million new cases of the active disease develop each year and three million people die from it [1]. Mycobacterial lymphadenitis comprises about 2% to 5% of all cases of TB and is more common among children, women and minorities, as well as in immunosuppressed patients, especially those with HIV [2,3]. The cervical lymph nodes are most frequently involved, followed by the mediastinal lymph nodes and the axillary lymph nodes. Disseminated mycobacterial lymphadenopathy, meanwhile, is extremely rare in non-HIV patients. As this kind of disseminated lymphadenopathy is of good prognosis due to antituberculous medication, diagnosis has to be confirmed by histologic and microbiological analyses. A possible misdiagnosis may lead to fatal complications for the patient [2,3].

We present the case of a 56-year-old Caucasian Greek male, who was immunocompetent and HIV negative, but had a two-month history of recurring fever, loss of appetite and polylymphadenopathy. The patient was diagnosed with mycobacterial lymphadenopathy. This case highlights the need for additional scrutiny to reach this diagnosis.

## Case presentation

A 56-year-old Caucasian Greek male was referred to the emergency department of our hospital with a two-month history of recurring fever, loss of appetite and swelling in his neck.

Upon admission, he was obtunded and hyperthermic (axillary temperature, 38°C). The patient had a respiratory rate of 20 breaths/min and a heart rate of 100 beats/min. He had no skin lesions. Upon physical examination, he was found to have bilateral cervical, axillary and inguinal lymphadenopathy. His lymph nodes were tenacious, unmovable and tender, and measured from 1.0 × 1.5 cm to 3.0 × 3.5 cm. A respiratory examination revealed mild bilateral inspiratory fine crackles. Chest X-rays showed right hilar lymphadenopathy. Laboratory data showed hemoglobin levels at 10.81 g/dl, a white blood cell count of 6,800 cells/mm^3 ^(70% neutrophils, 14% lymphocytes, 12% monocytes, 1% eosinophils) and a platelet count of 346.000 cells/mm^3^. His international normalized ratio was 1.11 and his activated partial thromboblastin time was 32.8 seconds.

The patient's erythrocyte sedimentation rate was 64 mm/h, and his C-reactive protein concentration was 9.97 mg/dl (normal range < 0.6 mg/dl). His total bilirubin was 0.13 mg/dl, his serum glutamic oxaloacetic transaminase (SGOT) was 113 IU/l, his serum glutamic pyruvic transaminase (SGPT) was 36 IU/l, his total protein was 7.69 mg/dl, and his creatinine phosphokinase (CPK) was 1777 IU/l. His renal function tests were within normal ranges, and an examination of his cerebrospinal fluid revealed no cells and its biochemical composition was normal. A Mantoux test was positive (20 mm), an arterial blood gas analysis while breathing room air showed pH 7.39, PaO_2_77 mmHg, PaCO_2 _40.6 mmHg, and HCO_3 _25.1 mmol/l. A protein electrophoresis did not show any monoclonal spike. Serolologic tests for hepatitis A, B, and C viruses and HIV were negative.

**Figure 1:**
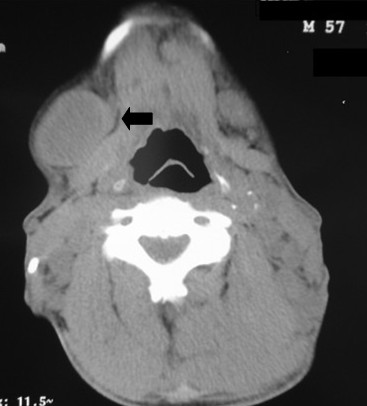
**The computed tomography scan of the neck shows an enlarged right submaxillary lymph node**.

**Figure 2:**
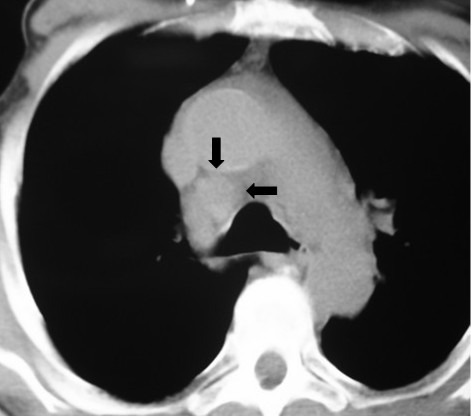
**A computed tomography scan of the chest showing enlarged paratracheal lymph nodes over the superior anterior mediastinum**.

**Figure 3:**
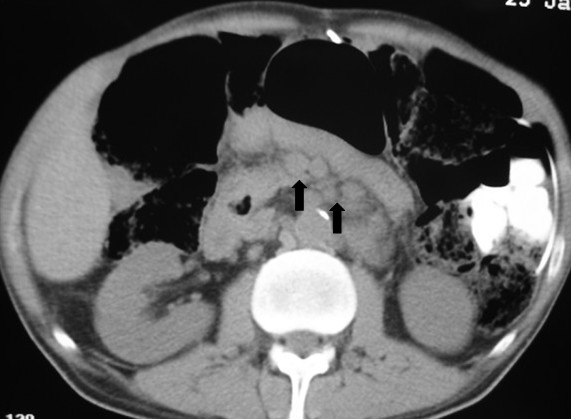
**An abdominal computed tomography scan showing para-aortic lymphadenopathy**.

An ultrasonography of the abdomen revealed hepatosplenomegaly and a computed tomography (CT) of the brain demonstrated no evidence of parenchymal lesions. A CT scan of the neck showed multiple enlarged lymph nodes in the right cervical chain, measuring up to 3.5 cm (Figure 1). A chest CT scan revealed numerous paratracheal lymph nodes over the superior-anterior mediastinum, measuring up to 2.8 cm (Figure 2). An abdominal CT showed lymph nodes in the lesser omentum, the mesentery, the anterior pararenal space, and the upper and lower para-aortic regions, measuring up to 2.5 cm (Figure 3).

Cervical lymph node and bone marrow biopsies were performed. The bone marrow biopsy was normocellular with mature hematopoietic elements. The lymph node was completely effaced with a lot of epithelioid cells and occasional Langhans giant cells, constituting well-defined granulomas with caseous necrosis, typical of TB. Immunohistochemically, the cells were negative for CD3, CD20, CD30, kappa and light chains. A Ziehl-Neelsen stain for acid-fast bacilli (AFB) was negative, but the culture grew *Mycobacterium tuberculosis *complex. The patient was started on antituberculous treatment with the standard four-drug regimen consisting of rifampin, pyrazinamide, ethambutol and isoniazid. After two months of treatment, he was symptom-free, with a prominent reduction in most lymph node swelling. The patient was started on a two-drug regimen of isoniazid and rifampin for seven months. At the end of the therapy, the patient had residual lymph nodes in the neck and in the mediastinum.

## Discussion

We present the case of a 56-year-old immunocompetent man with cervical, mediastinal, axillar, inguinal and abdominal lymphadenopathy, in whom tuberculous lymphadenopathy was diagnosed. Disseminated lymphadenopathy represents a challenge to a majority of clinicians and may be caused by a vast array of diseases, including mycobacterial infection.

TB is the foremost cause of death from a single infectious agent in humans. According to recent estimates, one person is newly infected with TB bacilli every second worldwide, and one third of the global population is currently infected with TB [1]. Poverty, HIV and drug resistance are major contributors to the resurging global TB epidemic. Approximately 95% of TB cases occur in developing countries. Approximately one in 14 new TB cases occurs in individuals infected with HIV, with 85% of these cases occurring in Africa [4,5].

While the primary site of infection in TB is the lungs, in up to 15% of cases an extrapulmonary site may produce the first symptoms [2]. Extrapulmonary TB is more common in children, women and minorities [6,7]. Lymphadenitis is the most common extrapulmonary presentation of TB. It occurs most commonly in the cervical region, representing 63% of all tuberculous lymphadenitis in one study of 1161 patients [2]. In the same study, the incidence of lymph node swelling detected in more than one site was 35.0%, whereas the incidence of inguinal lymph nodes was only 1.7% [2]. Although previously considered a childhood disease, lymphadenitis has a peak age of onset of 20 to 40 years [8,9]. Interestingly, in our case, a 56-year-old immunocompetent man who was HIV-negative had generalized lymphadenopathy including swollen inguinal lymph nodes.

The nodes in patients with mycobacterial lymhaphadenopathy are discrete, firm and non-tender. In time, a firm mass of matted nodes becomes visible. Hard, fixed nodes can be found in cancers and firm, rubbery nodes in lymphomas. If untreated, the tuberculous nodes become fluctuant and drain spontaneously with sinus tract formation. Patients with mycobacterial lymphadenopathy usually present with fever, night sweats and weight loss. Most patients have positive tuberculin skin test results and normal chest radiographs. An excisional biopsy of the lymph nodes with a histology, AFB stain and mycobacterial culture is the best diagnostic procedure [10]. The use of fine-needle aspirations in patients without HIV infections is highly variable [8]. A polymerase chain reaction for *Mycobacterium tuberculosis *of the fine needle aspiration specimen enhances test sensitivity [11].

A 6- to 9-month regimen (2 months of isoniazid, rifampin, pyrazinamide, and ethambutol, followed by 4 to 7 months of isoniazid and rifampin), is recommended as initial therapy for all forms of extrapulmonary TB, unless the organisms are known or strongly suspected to be resistant to first-line drugs [12]. During antituberculous therapy, affected nodes may enlarge or new nodes may appear, representing an immune response to killed mycobacteria. This phenomenon may lead to doubts about the accuracy of the diagnosis among inexperienced observers; however, since the enlargement of the lymph nodes during therapy is not unusual, it does not represent a sign of treatment failure [13]. Lymph node excision in the *M. tuberculosis *complex disease is not usually indicated. Relapse rates of up to 3.5% have been reported in patients treated for TB lymphadenitis [14]. A minority of adequately treated patients will have residual lymph nodes present at the end of the planned treatment course.

## Conclusion

Disseminated lymphadenopathy represents a major diagnostic problem. The differential diagnosis in an immunocompetent adult includes sarcoidosis, metastatic disease, lymphoma and, although rarely present, TB. Generalized lymph node involvement is uncommon in TB. In view of its relatively rare nature but accurate prognosis due to antituberculous medication, it is important to distinguish tuberculous lymphadenopathy in adults from other causes of generalized lymphadenopathy.

## Abbreviations

AFB: acid-fast bacilli; CPK: creatinine phosphokinase; CT: computed tomography; SGOT: glutamic oxaloacetic transaminase; SGPT: serum glutamic pyruvic transaminase; TB: tuberculosis.

## Consent

Written informed consent was obtained from the patient for publication of this case report and any accompanying images. A copy of the written consent is available for review by the Editor-in-Chief of this journal.

## Competing interests

The authors declare that they have no competing interests.

## Authors' contributions

IG and MP were involved in patient care and were jointly responsible for writing and revising the manuscript. AK was involved in patient care. MI performed the histological examination of the lesion. KK provided supervision in the writing of the manuscript. KG provided the overall editorial and clinical supervision. All authors read and approved the final manuscript.
